# *In Vitro* Neutralization of Low Dose Inocula at Physiological Concentrations of a Monoclonal Antibody Which Protects Macaques against SHIV Challenge

**DOI:** 10.1371/journal.pone.0072702

**Published:** 2013-08-16

**Authors:** David Davis, Wim Koornstra, Zahra Fagrouch, Ernst J. Verschoor, Jonathan L. Heeney, Willy M. J. M. Bogers

**Affiliations:** 1 Department of Virology, Biomedical Primate Research Centre, Rijswijk, The Netherlands; 2 Laboratory of Viral Zoonotics, University of Cambridge, Cambridge, United Kingdom; University of Amsterdam, Netherlands

## Abstract

**Background:**

Passive transfer of antibodies can be protective in the simian human immunodeficiency virus (SHIV) – rhesus macaque challenge model. The human monoclonal antibody IgG1 b12 neutralizes human immunodeficiency type 1 (HIV-1) *in vitro* and protects against challenge by SHIV. Our hypothesis is that neutralizing antibodies can only completely inactivate a relatively small number of infectious virus.

**Methods And Findings:**

We have used GHOST cell assays to quantify individual infectious events with HIV-1_SF162_ and its SHIV derivatives: the relatively neutralization sensitive SHIV_SF162P4_ isolate and the more resistant SHIV_SF162P3_. A plot of the number of fluorescent GHOST cells with increasing HIV-1_SF162_ dose is not linear. It is likely that with high-dose inocula, infection with multiple virus produces additive fluorescence in individual cells. In studies of the neutralization kinetics of IgG1 b12 against these isolates, events during the absorption phase of the assay, as well as the incubation phase, determine the level of neutralization. It is possible that complete inactivation of a virus is limited to the time it is exposed on the cell surface. Assays can be modified so that neutralization of these very low doses of virus can be quantified. A higher concentration of antibody is required to neutralize the same dose of resistant SHIV_SF162P3_ than the sensitive SHIV_SF162P4_. In the absence of selection during passage, the density of the CCR5 co-receptor on the GHOST cell surface is reduced. Changes in the CD4 : CCR5 density ratio influence neutralization.

**Conclusions:**

Low concentrations of IgG1 b12 completely inactivate small doses of the neutralization resistant SHIV _SF162P3_. Assays need to be modified to quantify this effect. Results from modified assays may predict protection following repeated low-dose shiv challenges in rhesus macaques. It should be possible to induce this level of antibody by vaccination so that modified assays could predict the outcome of human trials.

## Introduction

A correlate of protection would facilitate the development of a vaccine against human immunodeficiency type 1 (HIV-1). A likely candidate is neutralization [1,2] since monoclonal antibodies alone can protect rhesus macaques challenged with simian human immunodeficiency virus (SHIV) [3–6]. SHIV engineered with HIV-1_SF162_ envelope glycoproteins [7] is particularly relevant since it can infect mucosally and uses CC-chemokine receptor (CCR5) as a co-receptor to enter cells in line with the majority of natural transmission events [8]. Passage of SHIV_SF162_ through rhesus macaques produces variants which have a range of pathogenicities and neutralization sensitivities [9–12]. The human monoclonal antibody IgG1 b12 [13] can prevent SHIV_SF162_ infection of rhesus macaques [14–17]. However, the dose of antibody required for complete protection is so high that it is likely to be beyond that which can be achieved by immunization [14,15,17]. A pragmatic goal for vaccination would be to induce a combination of cell-mediated immunity and neutralizing antibodies which could control the replication of virus within an infected individual [14,15,17,18].

HIV-1_SF162_ was isolated from cerebrospinal fluid of a patient with *Toxoplasma* [19]. It is subtype B. It is monocytotropic and does not replicate in continuous cell lines. It was originally classified into the neutralization resistant group, relative to other HIV-1 isolated from peripheral blood mononuclear cells (PBMCs) of patients in San Francisco [20–24]. This classification was later changed to relatively neutralization sensitive. The *tat*, *rev*, *vpu* and *env* genes of HIV-1_SF162_ were transferred to an infectious clone of simian immunodeficiency virus (SIV_mac239_) [7]. Infectious virus was produced in cell culture and passaged, intravenously, four times through juvenile rhesus macaques [12]. The resulting SHIV_SF162P4_ still exclusively used CCR5 as its co-receptor [8]. While the envelope glycoprotein accumulated mutations in individual virus, the consensus sequence of the polymorphic mixture of variants showed no change from the parental HIV-1_SF162_ clone [25]. One of the macaques at the third passage became chronically infected and subsequently developed simian acquired immunodeficiency syndrome (SAIDS) [26]. Virus, SHIV_SF162P3_, was isolated from its lymph nodes [27]. An infectious molecular clone of SHIV_SF162P3_ has been produced [28].

The SHIV_SF162_ variants are infectious for adult rhesus macaques by the oral, intravenous, intra-vaginal and intra-rectal routes [14–17,26–37]. They have been used in passive transfer and immunization studies. Both variants are pathogenic inducing a range of clinical conditions from rapid progression, without seroconversion, through longer-term non-progression to chronic infection with SAIDS one to two years after infection [11,27,32]. While most rhesus macaques are able to clear their plasma viremia, virus can still be isolated from peripheral lymphocytes over extended periods [7,8]. There is an acute, transient reduction in peripheral CD4 positive lymphocytes followed by a recovery and subsequently, a gradual reduction in their number [8,27,36]. SHIV_SF162_ infects the gut-associated lymphoid tissue producing large reductions in CD4 positive lymphocyte numbers [8]. SHIV_SF162P3_ contains several variants: the major variant has 14 amino acid differences in its external envelope glycoprotein (gp120) and a further two and four in the external and internal parts of the transmembrane envelope glycoprotein (gp41) relative to the parental clone. It is considered to be relatively resistant to neutralization [9,10].

IgG1 b12 is a human monoclonal antibody [13]. It recognizes an epitope which overlaps the CD4-binding site of the HIV-1 envelope glycoprotein and can neutralize a wide range of isolates from multiple subtypes [38,39]. Doses of 25 mg/kg IgG1 b12 were required to fully protect rhesus macaques against intra-vaginal challenge with 300 tissue culture infectious doses (TCID_50_) of SHIV_SF162P4_. Monitoring the activity of plasma taken at the time of challenge indicated that a hundredfold more antibody is required to protect *in vivo* than to neutralize *in vitro* [17]. Lower antibody concentrations reduced and delayed the *in vivo* peak plasma viremia. Challenge of macaques with high doses of SHIV is required for experimental challenges, so that all control macaques become infected, but may not represent the conditions prevailing in natural transmission events. SHIV_SF162P3_ can be used in repeated low dose challenges of rhesus macaques which may better represent natural transmission [36,40]. Lower concentrations of antibody may be protective against reduced doses of virus. In a repeated low dose (10 TCID_50_), intra-vaginal challenge model with rhesus macaques, passively transferred with doses of 1 mg/kg IgG1 b12, the number of exposures required for infection with SHIV_SF162P3_ was increased and the peak viral load was also reduced [15].

GHOST cells are human osteosarcoma cells which have been engineered to express human CD4 and CCR5 molecules [41]. They fluoresce when infected with a primate lentivirus and can be used to quantify infectious events in neutralization assays using a fluorescence activated cell scanner (FACS) [42]. Previous studies using human PBMCs as target cells have indicated that the kinetics of HIV-1 neutralization by monoclonal antibodies does not follow the traditional pattern [43]. The aim of the present study was to investigate the kinetics of neutralization with a combination of virus and antibody which is known to be protective *in vivo*. GHOST cell assays are used to offer greater precision in quantifying neutralization [44].

## Results

### Relation of the number of fluorescent cells to the virus inoculum

**Figure 1:**
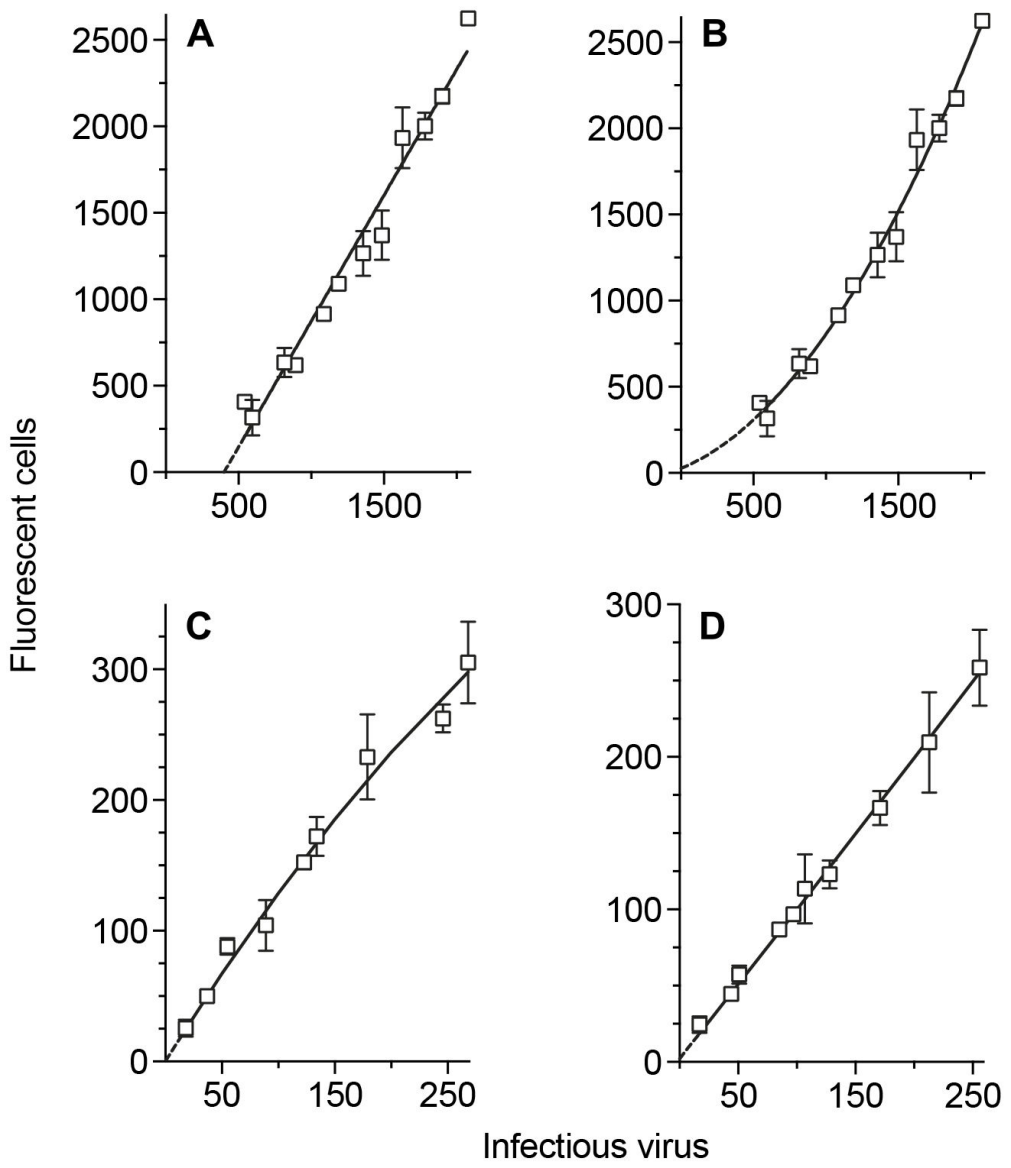
Dose–response plots of HIV-1 _SF162_ and SHIV variants on GHOST cells. GHOST cell cultures were exposed to different doses of virus, plotted on the x-axis (= horizontal). The number of cells which fluoresce after infection is plotted on the y-axis (= vertical). **A**. Linear regression of HIV-1 _SF162_ - infected cultures: y = 1.457 ± 0.066 x -584.3 ± 89.86. **B**. Fitting of data to second-order (quadratic) equation: y = 4.31 x 10^-4^ x^2^ + 0.347 x + 26.63. **C**. Quadratic plot of SHIV_SF162P4_ on GHOST cells: y = -1.03 x 10^-3^ x^2^ + 1.349 x + -4.17 x 10^-4^. **D**. Quadratic plot of SHIV_SF162P3_ on GHOST cells: y = -7.63 x 10^-5^ x^2^ + 0.963x + -4.077.

GHOST cells fluoresce when infected with HIV-1 or SHIV and can be used to quantify individual infectious events. A plot of virus dose against the number of fluorescent cells (Figure 1A) is linear with an r^2^ value of 0.9573 (p < 0.0001). However, the plot does not pass through the origin where there is no virus (x = 0 on the horizontal axis) and the number of fluorescent cells should indicate background levels. Instead, it cuts the horizontal axis at approximately 400 infectious doses. The gradient of the plot is 1.457 fluorescent cells for each infectious virus. Allowing for multiple virus infecting the same cell by a Poisson transformation of the data does not correct the displaced intercept.

If the same data are fitted to a second order (quadratic) plot which involves a term where the number of infectious virus is squared (Figure 1B) there is only a slight increase in the r^2^ value to 0.9729. However, the plot now cuts the vertical axis at 26 fluorescent cells (Figure 1B).

The SHIV stocks have lower titers of infectious virus and show approximately linear plots (Figure 1C, D)

### Neutralization of HIV-1_SF162_

**Figure 2:**
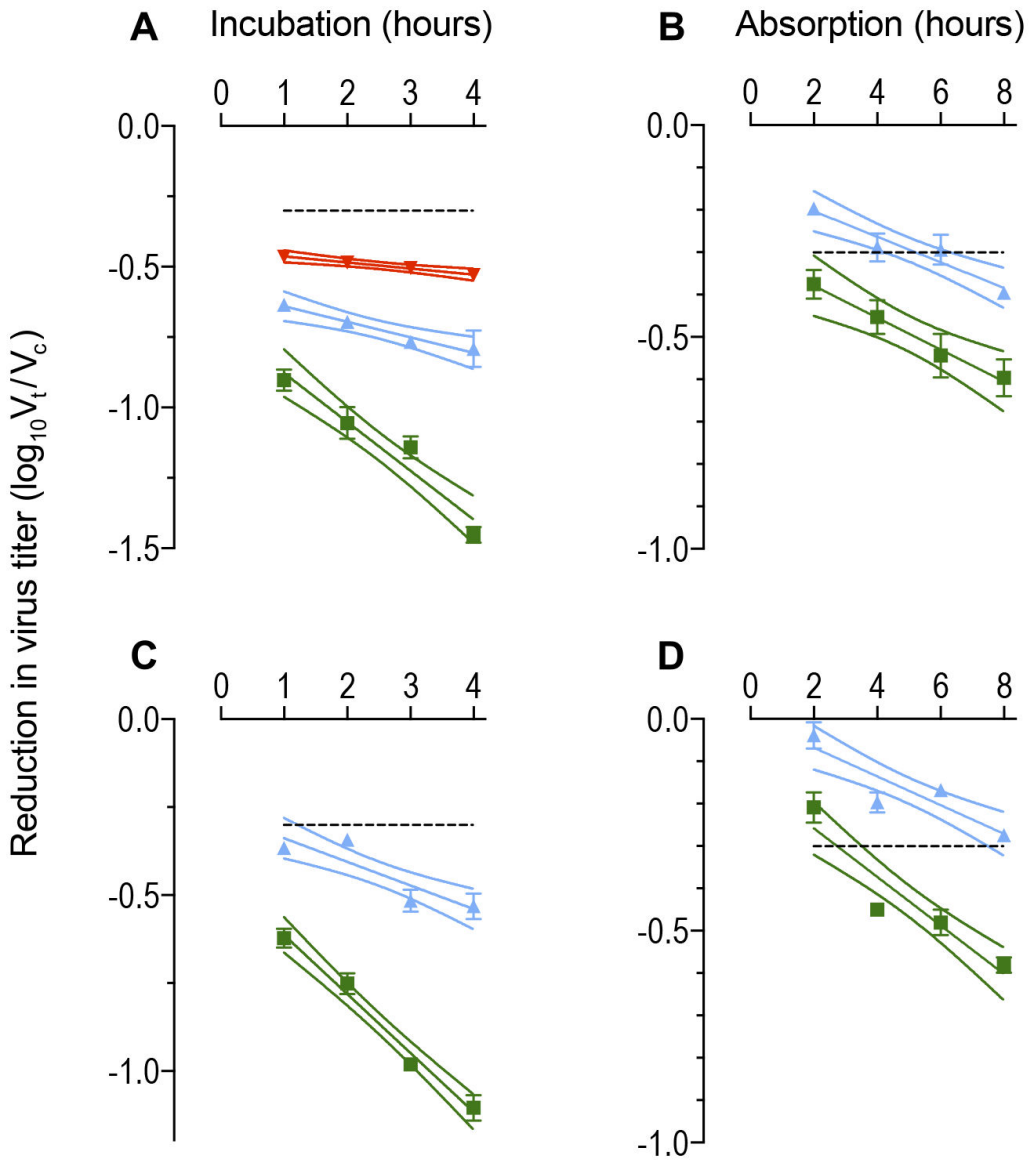
Reductions in infectious titer following exposure of HIV-1_SF162_ or SHIV_SF162P4_ to monoclonal antibody IgG 1 b12. Reductions in infectious virus are calculated as the ratio of the titer (V_t_) at time t for the virus exposed to antibody divided by the titer (V_c_) at the same time for control cultures without antibody. The ratio is transformed to log_10_ (V_t_ / V_c_). Incubation and absorption phases are measured in hours. Data are displayed as means with standard errors. Plots are regression lines with their 95% confidence band. Horizontal broken line represents 50% neutralization. Green: 1 µg/ml IgG1 b12; Blue: 0.4 µg/ml IgG1 b12; Red: 0.2 µg/ml IgG1 b12. Expected ratio of neutralization rates is the ratio of the antibody concentrations within an individual assay. **A**. Incubation plots of IgG1 b12 against HIV-1 _**SF162**_ (Ratios: Expected 1 µg/ml : 0.4 µg/ml = 2.5; The observed ratio of the gradients of the regression lines = 3.14; p < 0.0001; Expected 1 µg/ml : 0.2 µg/ml = 5; Observed: 8.03p < 0.0001; Expected 0.4 µg/ml : 0.2 µg/ml = 2; Observed = 2.56; p = 0.0251); **B**. absorption plots of IgG1 b12 against HIV-1 _**SF162**_ (Ratios Expected = 2.5; Observed = 1.25; p = 0.4879); C. Incubation plots of IgG1 b12 against SHIV **_SF162P4_** (Ratios: Expected = 2.50; Observed = 2.50; p < 0.0001); D. Absorption plots of IgG1 b12 against SHIV **_SF162P4_** (Ratios: Expected = 2.50; Observed = 1.69; p < 0.02773).

The primary isolate of HIV-1_SF162_ shows exponential neutralization (equal proportions of virus are inactivated per unit of time) following exposure to the monoclonal antibody IgG1 b12 (Figure 2A). The neutralization rate is between log_10_ 0.158 and log_10_ 0.223 infectious doses per hour with 1 µg/ml of antibody over a range of virus doses (222-821 infectious doses) and cell passage numbers (11-16). The ratios of the neutralization rates at two concentrations of antibody during the incubation phase (2.01-3.14) were close to the expected value of 2.50 (Table 1) derived from the antibody concentrations. Nonetheless, it is apparent that if the incubation plots are extrapolated back to zero time (= the intercept where the plot crosses the vertical or y-axis) they do not pass through the origin (point 0, 0 where the vertical and horizontal axes cross): there is significant neutralization (> 50%) without any incubation. As the virus is slow to bind to the target cells, this neutralization may be the result of antibody binding to free virions in the supernatant above the target cells. Alternatively, the presence of cells may be obligatory and events following the exposure of virus or virus-antibody complexes to targets may determine the eventual extent of neutralization.

**Table 1:** Neutralization of HIV-1 _SF162_ and SHIV variants by human IgG1 b12 monoclonal antibody in GHOST cell assays.

		**Antibody concentration**	
**Cell passage**	**Virus inoculum**	**1 µg/ml**	**0.4 µg/ml**	**Ratio**	Significance^*^
HIV-1 _SF162_ (Incubation rate)					
11	821	- 0.173 ± 0.021^§^	-0.055 ± 0.014	3.14	< 0.0001
12	222	-0.223 ± 0.037	-0.077 ± 0.029	2.90	0.006488
16	389	-0.158 ± 0.032	-0.079 ± 0.014	2.01	0.02993
HIV-1 _SF162_ (Absorption rate)					
11	311	-0.039 ± 0.009	-0.026 ± 0.009	1.51	0.3021
18	442	-0.038 ± 0.009	-0.030 ± 0.006	1.25	0.4879
SHIV _SF162P4_ (Incubation rate)					
11	924	-0.168 ± 0.013	-0.067 ± 0.014	2.50	< 0.0001
16	653	-0.115 ± 0.007	-0.025 ± 0.006	4.59	< 0.0001
SHIV _SF162P4_ (Absorption rate)					
18	447	-0.060 ± 0.006	-0.040 ± 0.006	1.52	0.02927
11	1,024	-0.057 ± 0.008	-0.034 ± 0.006	1.69	0.02773
**Cell passage**	**Virus inoculum**	**1 µg/ml**	**2.5 µg/ml**	**Ratio**	**Significance**
SHIV _SF162P3_ (Incubation rate)					
11	747	-0.049 ± 0.014	-0.094 ± 0.015	1.91	0.03782

^*^Significance : ratio of neutralization rates at different antibody concentrations

^§^log_10_ infectious doses per hour

Indeed, the loss of virus infectivity is also exponential following exposure of the virus-antibody mixture to the target cells (Figure 2B). The neutralization rate during the absorption phase is log_10_ 0.039 infectious doses per hour with 1 µg/ml of antibody (Table 1). There was no statistical significance between the neutralization rates with different concentrations of antibody during the absorption phase so that their ratio was reduced below the expected value (Table 1).

### Neutralization of SHIV variants of HIV-1_SF162_

The neutralization sensitive SHIV_SF162P4_ shows exponential loss of virus titer following exposure to IgG1 b12 (Figure 2C). The rate (a reduction of log_10_ 0.115 - log_10_ 0.168 infectious doses per hour with 1 µg/ml of antibody) is lower than the parental HIV-1_SF162_ while the ratio of the rates at different antibody concentrations is statistically significant and close to the expected value (Table 1). Loss of virus titer is also exponential during the absorption phase (Figure 2D). The absorption phase neutralization rate (log_10_ 0.060 infectious doses per hour with 1 µg/ml of antibody) is higher than that with the parental isolate (Table 1). The mean ratio of the absorption neutralization rates at different antibody concentrations (1.61) reaches statistical significance (Table 1).

The loss of infectivity of the neutralization resistant SHIV_SF162P3_ variant following exposure to IgG1 b12 (log_10_ 0.049 infectious doses per hour with 1 µg/ml of antibody) is reduced relative to both SHIV_SF162P4_ and the parental isolate (Table 1). The ratio of the rates at two antibody concentrations (1.91) reaches statistical significance (Table 1). Titers of the SHIV_SF162P3_ stock were insufficient to estimate neutralization rates during the absorption phase.

### Combined incubation and absorption plots

**Figure 3:**
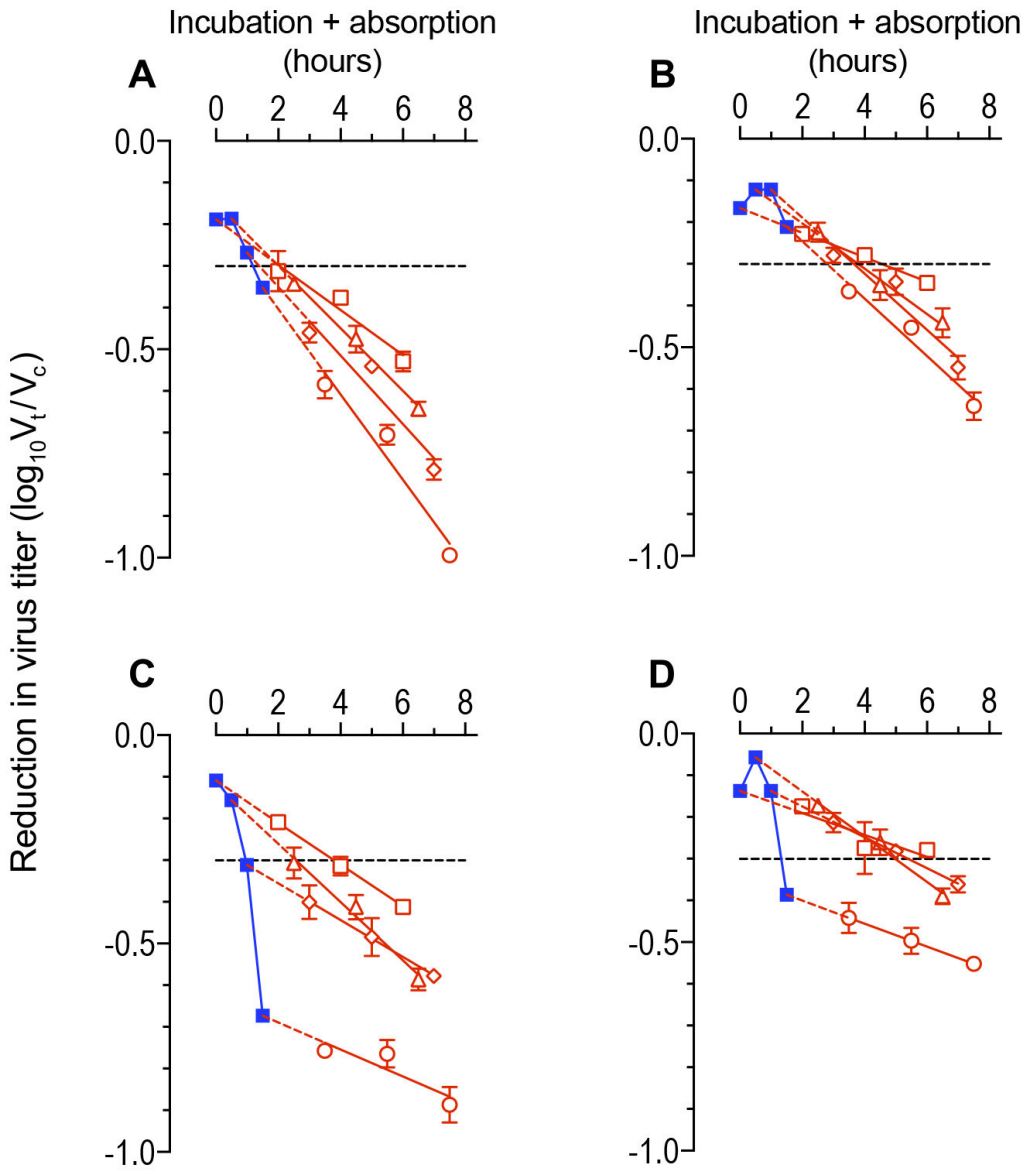
Incubation plus absorption plots of HIV-1 _SF162_ neutralization with human monoclonal antibody IgG1 b12. Reductions in infectious virus are calculated as the ratio of the titer (V_t_) at time t for the virus exposed to antibody divided by the titer (V_c_) at the same time for control cultures. The ratio is transformed to log_10_ (V_t_ / V_c_) and plotted on the y axis (= vertical). Incubation and absorption phases are measured in hours and plotted on the x-axis (= horizontal). Data are displayed as means with standard errors. Broken horizontal line represents 50% neutralization. Red triangles, diamonds and discs: linear regression lines for absorption plots following incubation for different time intervals. Intercepts determined (dotted red lines) giving reduction in virus titer when absorption is zero (≡ end of incubation phase) and plotted as solid blue squares. Second-order (quadratic) lines fitted to the incubation (= blue) plot. **A**. 1 µg/milliliter IgG1 b12 vs inocula of 1,955 infectious doses: incubation plot (blue) is y = -0.086 x^2^ + 0.014 x -0.185; r^2^ = 0.9822. **B**. 0.4 µg / ml IgG1 b12 vs inocula of 1,955 infectious doses: incubation plot (blue) is y = -0.134 x^2^ + 0.174 x -0.169; r^2^ = 0.9818. **C**. 1 µg/milliliter IgG1 b12 vs inocula of 592 infectious doses: incubation plot (blue) is y = -0.315 x^2^ + 0.102 x -0.113; r^2^ = 0.9976. **D**. 0.4 µg / ml IgG1 b12 vs inocula of 592 infectious doses: incubation plot (blue) is y = -0.329x^2^ + 0.329x -0.138; r^2^ = 1.000.

An absorption phase is required for any neutralization assay but by extrapolating the plots back, the points where they cross the vertical axis can give a measure of the neutralization at zero time of absorption which also corresponds with the end of the incubation phase. Plots where both incubation and absorption phases were varied (Figure 3) indicate that there was a delay before inactivation of free virions enters its exponential phase.

### Neutralization at low doses of HIV-1_SF162_

Recognition that events during both the absorption and incubation phases in HIV-1 neutralization assays produce significant effects leads to further conjectures. Firstly, only a limited number of viruses may be completely inactivated before the target cells remove them or their complexes from the mixture. This contrasts with the proportion of virus which is expected to be inactivated in the reversible reaction between antibody and free virions. It is possible that low doses of virus may be completely inactivated by much lower concentrations of antibody than would be required to give significant neutralization against high doses of the virus.

**Figure 4:**
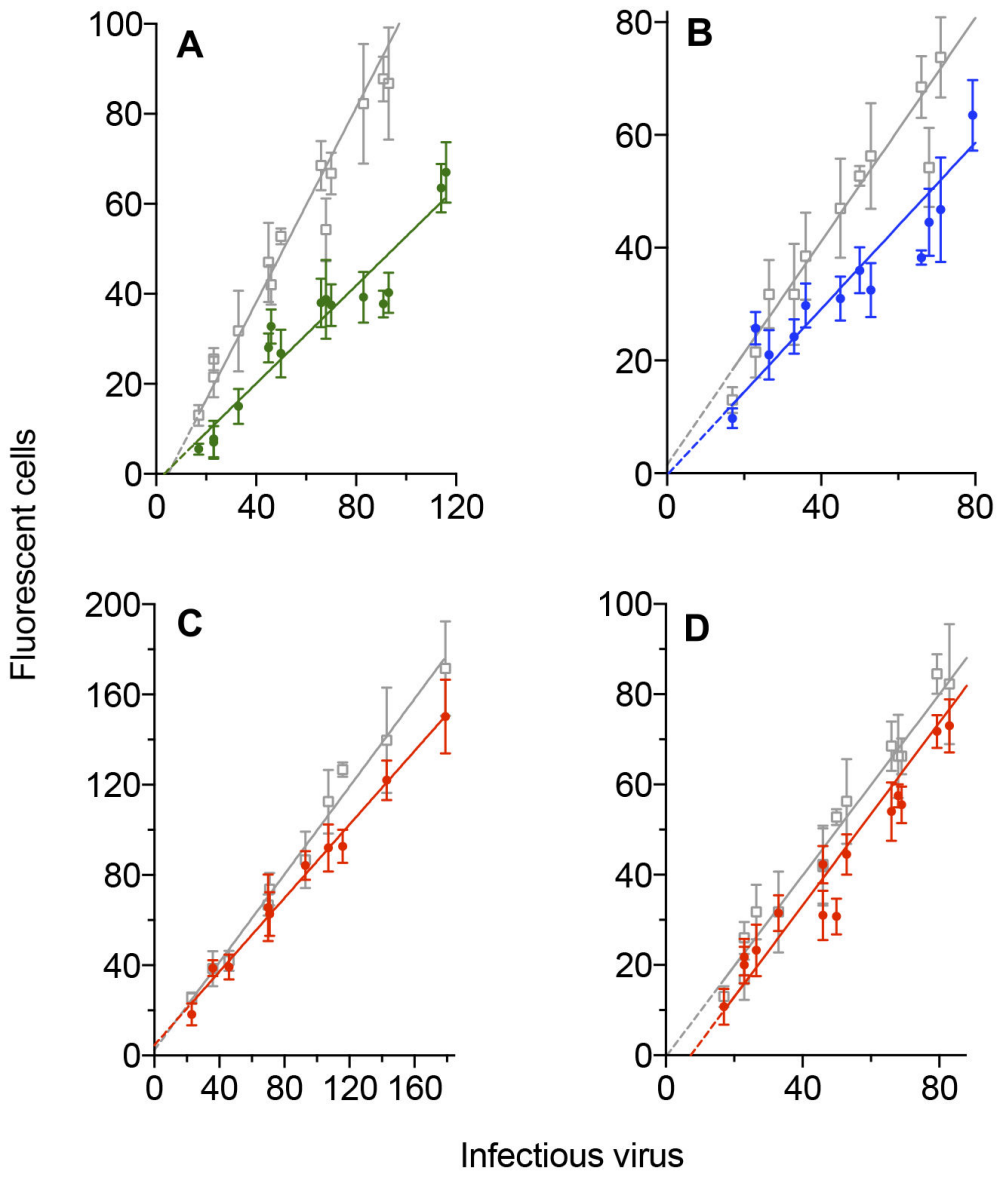
Comparison of linear regression and fitted second-order plots of reductions in infectious virus in HIV-1 _SF162_ - GHOST cell neutralization assays. Low doses of the relatively neutralization sensitive HIV-1 _SF162_ isolate were incubated at 37^0^C for four hours with concentrations of the human monoclonal antibody IgG1 b12. The mixture was then added to GHOST cells and allowed to absorb for 24 hours. The cells were washed and cultured for a further 24 hours (= 4/24/2 assays). Four duplicate cultures were used for each point within a replicate. Parameters are given as means with their standard errors. Regression lines with the formula y = mx + c where y is the number of fluorescent cells plotted on the vertical axis and x is the dose of virus along the horizontal axis. **A**. Gray: three control replicates where cells were cultured without monoclonal antibody: m = 1.081 ± 0.063; c = -5.187 ± 4.375; green: three replicates where virus were incubated with 0.125 µg/ml IgG1 b12 : m = 0.545 ± 0.042; c = -1.731 ± 2.938. **B**. Gray: seven control replicates where cells were cultured without monoclonal antibody: m = 1.003 ± 0.037 ; c = -0.300 ± 3.086; blue: seven replicates where virus were incubated with 0.05 µg/ml IgG1 b12 : m = 0.737 ± 0.028; c = -0.409 ± 2.350. **C**. Gray: two control replicates where cells from early passages were cultured without monoclonal antibody: m = 0.973 ± 0.076; c = 2.409 ± 7.608; red: two replicates where virus were incubated with 0.02 µg/ml IgG1 b12: m = 0.815 ± 0.059; c = -4.545 ± 5.928; D. Gray: four control replicates where cells from later passages were cultured without monoclonal antibody: m = 1.003 ± 0.046; c = -0.301 ± 3.402; red: four replicates where virus were incubated with 0.02 µg/ml IgG1 b12: m = 1.013 ± 0.036; c = -7.313 ± 2.719. Dotted lines are extrapolations to the axes using the formula of the regression / fitted lines. Some data points have been excluded and axes truncated to improve clarity and magnify the situation around the origin.

At the low doses of virus used in these assays the number of cells which fluoresce following exposure to HIV-1_SF162_ increases linearly with virus dose (Figure 4). Plots have a gradient close to one and pass near the origin: fitting the data to a quadratic equation, where an x^2^ term is included, does not increase the regression coefficient at the doses of virus used. Neutralization of the virus in 4/24/2 assays can be quantified by reductions in the gradients of their plots: 50% neutralization with 0.125 µg/ml IgG1 b12 (Figure 4A); 27% neutralization with 0.05 µg/ml IgG1 b12 (Figure 4B). Linear regression analysis in each of the seven replicate assays indicates that neutralization of HIV-1_SF162_ by 0.05 µg/ml IgG1 b12 reaches statistical significance (p < 0.05) in either its gradient or intercept. In early passage cultures with 0.02 µg/ml IgG1 b12, there is 16% neutralization (Figure 4C). However, with target cells from later passages (Figure 4D), the plots are parallel (equivalent to -1.0% neutralization calculated from their gradients). If the individual virus doses are used for calculation, neutralization increases from 8% with 80 infectious doses to 100% with inocula below seven infectious doses. There is an interval of seven infectious doses between the points where the control and antibody treated plots cut the horizontal axis (Figure 4D).

### The surface expression of CCR5 falls as GHOST cells are passaged without selection

**Figure 5:**
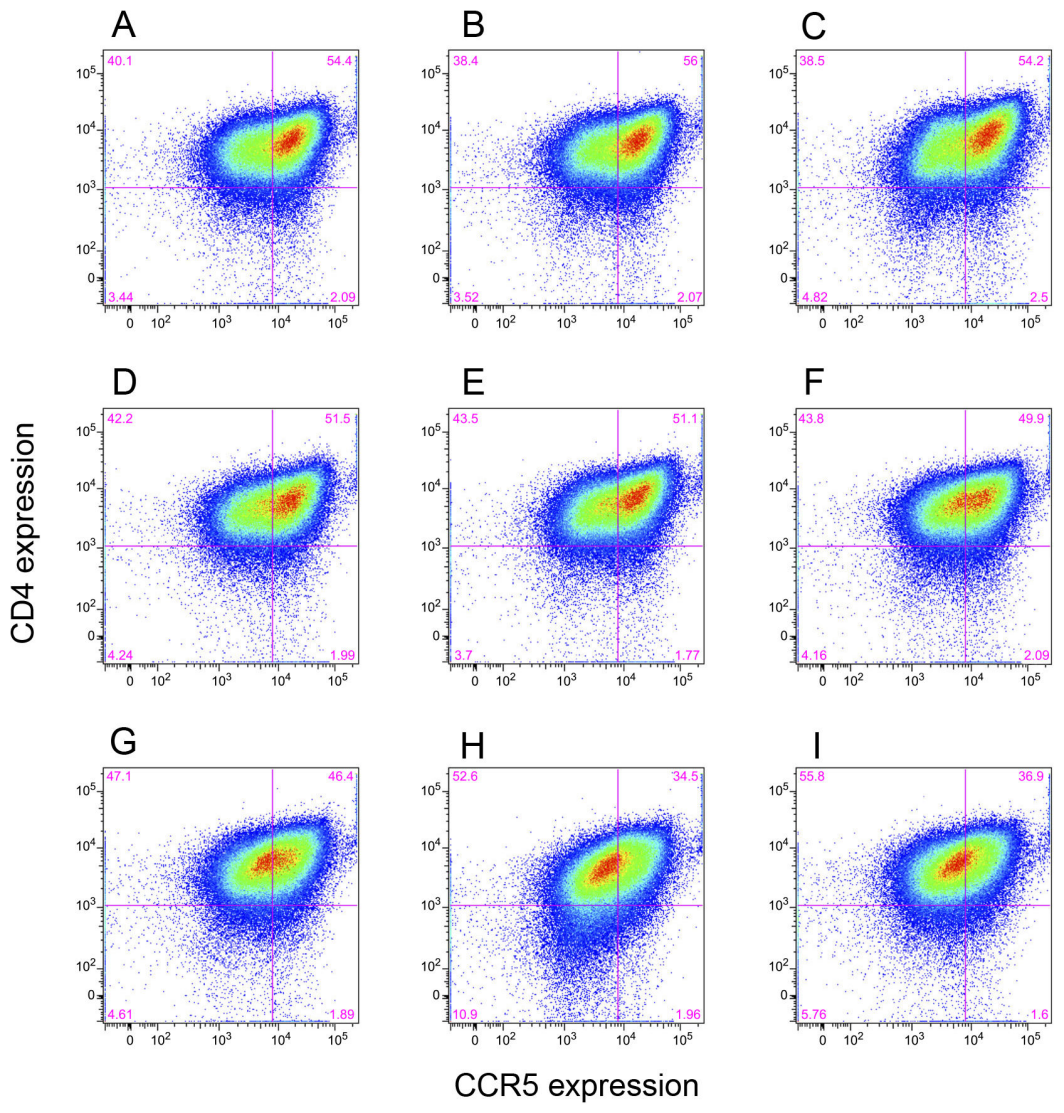
FACS analysis of Hi5 GHOST cells at different passage levels. Fluorescent intensities for CD4 are plotted on the vertical axis and CCR5 horizontally. Upper left quadrant quantifies target cells with low levels of CCR5 and the upper right those with higher levels. **A**. Passage 7; **B**. Passage 9; **C**. Passage 11; **D**. Passage 13; **E**. Passage 15; **F**. Passage 17; **G**. Passage 19; **H**. Passage 21; **I**. Passage 23.

**Figure 6:**
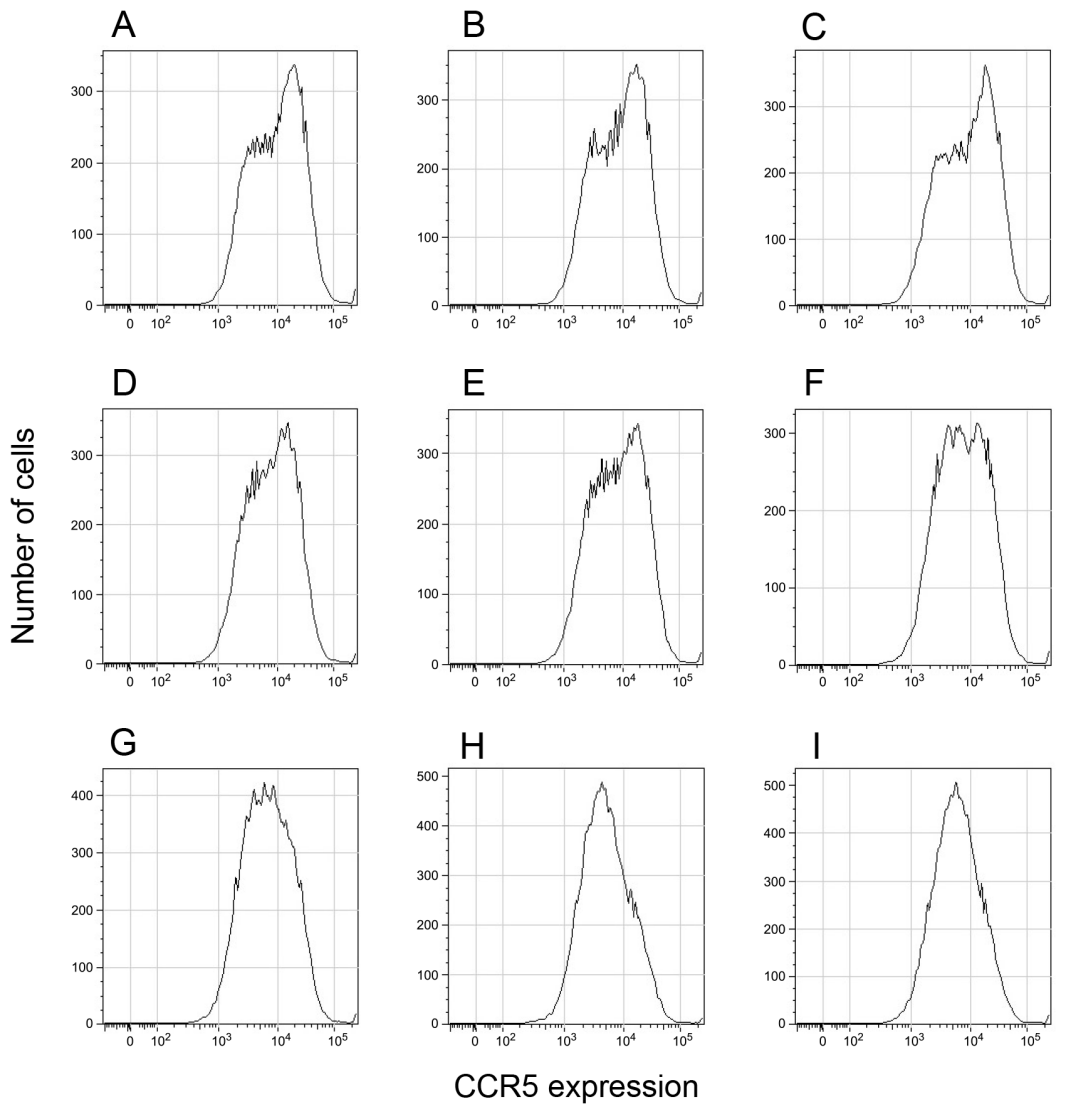
FACS analysis of Hi5 GHOST cells at different passage levels. Fluorescent intensities are plotted on the horizontal axis and the number of cells on the vertical. **A**. Passage 7; **B**. Passage 9; **C**. Passage 11; **D**. Passage 13; **E**. Passage 15; **F**. Passage 17; **G**. Passage 19; **H**. Passage 21; **I**. Passage 23.

The observation that neutralization is influenced by the passage number indicates that some changes must be occurring in the target cells. One possibility is that the cultures are infested with mycoplasma and their levels may be increasing following passage. No mycoplasma was detected in the GHOST cells at any passage level. Alternatively, the changes may be related to the practice of passaging the Hi5 CCR5 GHOST cells in the absence of any selective pressure related to co-receptor expression. While the surface expression of the primary HIV receptor, CD4, is maintained that of the CCR5 co-receptor declines as the passage number increases (Figure 5). At early passage levels the main fluorescence peak represents cells with a high surface density of CCR5. However, there are also cells with a lower level of the co-receptor. The ratio of the high : low density cells gradually reverses on passage without artificial selection until the lower density cells predominate (Figure 6).

### Neutralization at low doses of SHIV_SF162P4_

**Figure 7:**
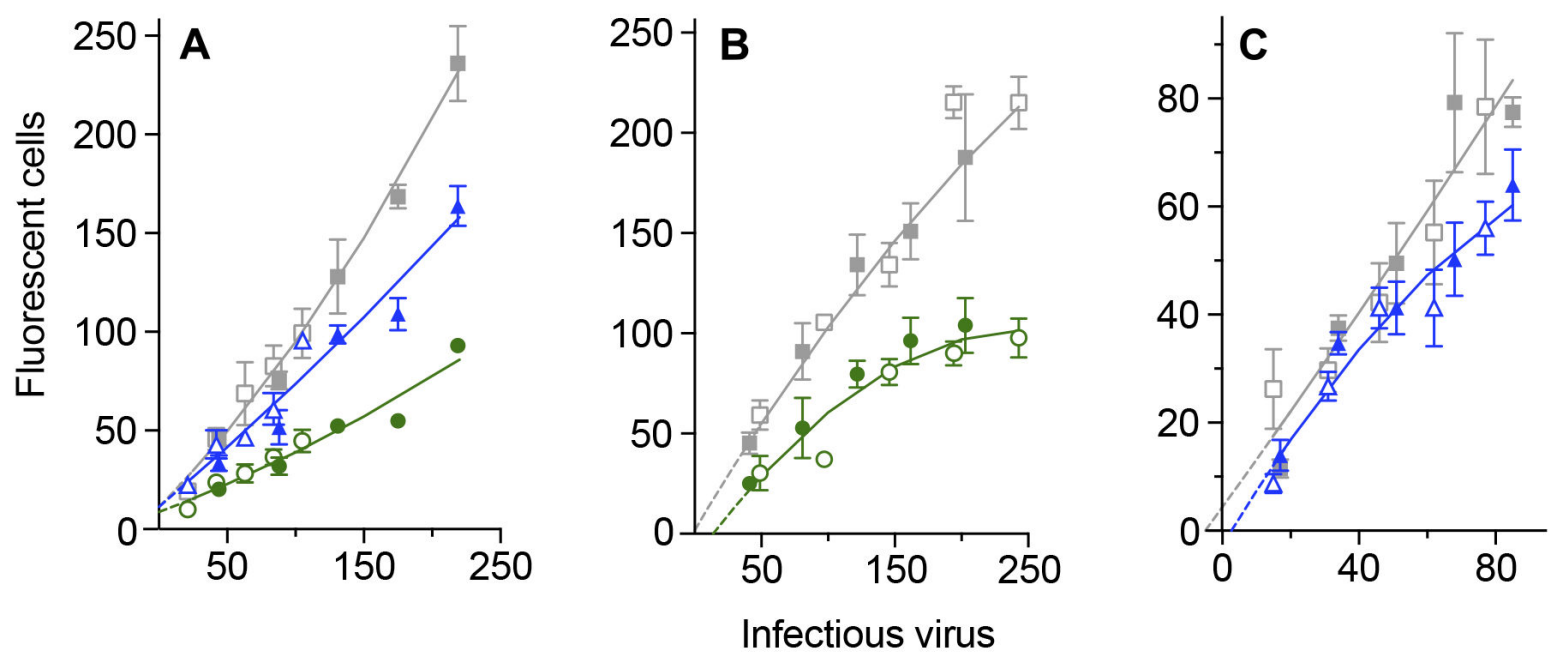
Neutralization of low doses of SHIV_SF162P4_ in assays with GHOST cells at different passage levels. Low doses of the relatively neutralization sensitive SHIV _SF162P4_ isolate were incubated at 37^0^C for four hours with concentrations of the human monoclonal antibody IgG1 b12. The mixture was then added to GHOST cells and allowed to absorb for 24 hours. The cells were washed and cultured for a further 24 hours (= 4/24/2 assays). Four duplicate cultures were used for each point within a replicate. Parameters are given as means with their standard errors. Dotted lines are extrapolations to the horizontal axis calculated from the quadratic plots. Open symbols: replicate 1; closed symbols: replicate 2. Data are fitted to a second-order (quadratic) equation **A**. SHIV_SF162P4_ exposed to GHOST cells from passage 9. Gray: control cultures where virus were incubated without monoclonal antibody: y = + 0.00143 x^2^ + 0.693 x + 11.28; blue: Virus pre-incubated with 0.1 µg/ml IgG1 b12: y = + 0.000386 x^2^ + 0.582 x + 11.47; green: Virus pre-incubated with 0.25 µg/ml IgG1 b12: y = + 0.000406 x^2^ + 0.261 x + 8.767. **B**. SHIV_SF162P4_ exposed to GHOST cells from passage 15. Gray: control cultures where virus were incubated without monoclonal antibody: y = -0.000998 x^2^ + 1.112 x + 1.795. green: Virus pre-incubated with 0.25 µg/ml IgG1 b12: y = -0.00184 x^2^ + 0.915 x -12.53. Interval between points where plots cut x-axis: 15.7 infectious virus. **C**. SHIV_SF162P4_ exposed to GHOST cells from passage 15. Gray: control cultures where virus were incubated without monoclonal antibody: y = 0.000625 x^2^ + 0.877 x + 4.278. blue: where cultures were exposed to virus pre-incubated with 0.1 µg/ml IgG1 b12: y = -0.00374x^2^ + 1.062x -2.941. Interval between points where plots cut x-axis: 7.68 infectious virus.

The neutralization sensitivity of SHIV_SF162P4_ is close to that of HIV-1 _SF162_ (Table 1 and compare Figures 2 and 7). A quadratic equation, where an x^2^ (the number of infectious virus is squared) component is introduced to the formula, offers a better fit, in terms of increased regression coefficients, to the data than linear regression. When target cells are from early passage levels, the neutralization plots show decreasing numbers of fluorescent cells as antibody concentration is increased (Figure 7A). The points where the plots cross the vertical axis are positive and close together.

With cells which have been further passaged, the points where the plots cross the horizontal axis are separated: an interval of 16 infectious doses for 0.25 µg/ml IgG1 b12 (Figure 7B) and eight with 0.10 µg/ml IgG1 b12 (Figure 7C).

### Neutralization at low doses of SHIV_SF162P3_

**Figure 8:**
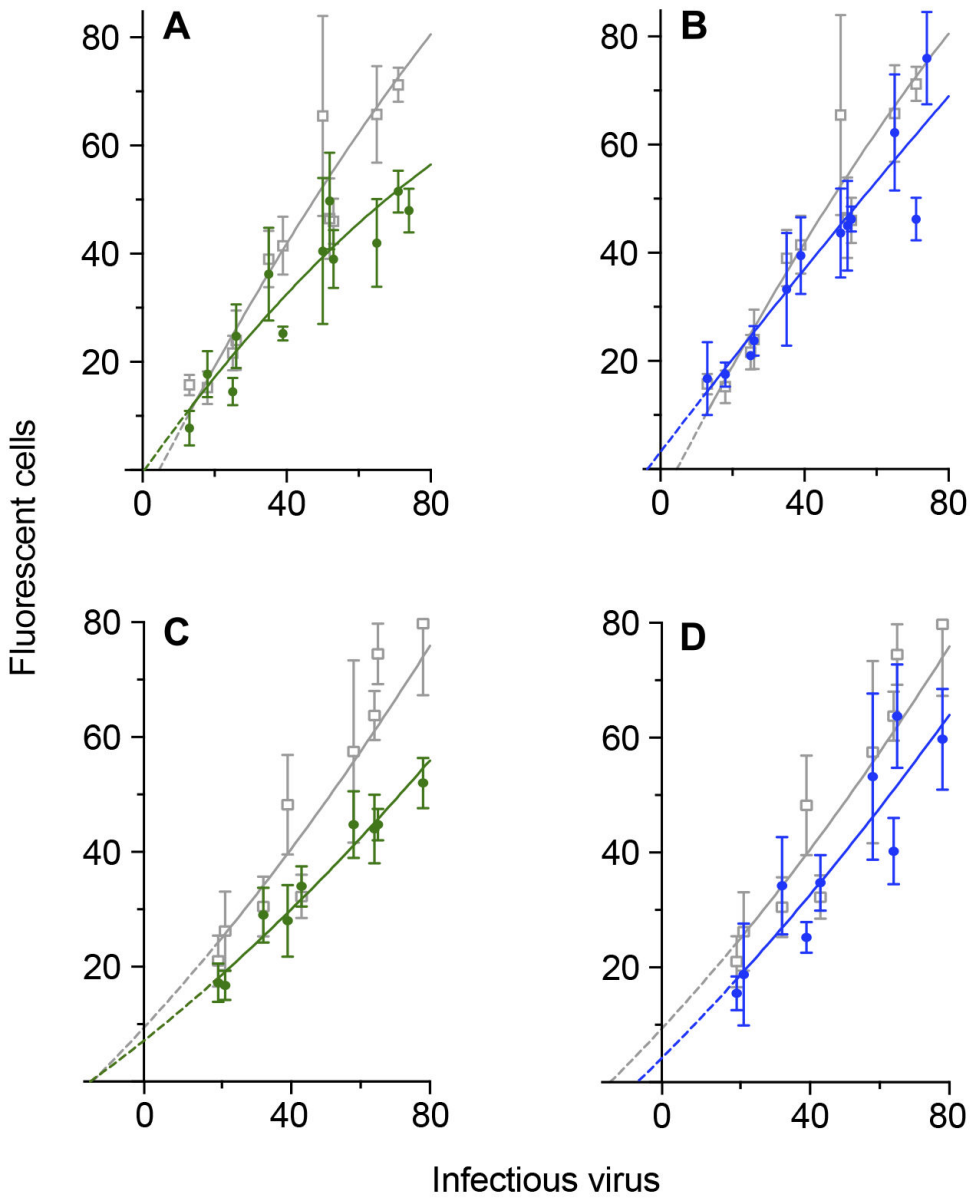
Neutralization of low doses of SHIV_SF162P3_ in assays with GHOST cells at different passage levels. Low doses of the relatively neutralization resistant SHIV _SF162P3_ isolate were incubated at 37^0^C for four hours with concentrations of the human monoclonal antibody IgG1 b12. The mixture was then added to GHOST cells and allowed to absorb for 24 hours. The cells were washed and cultured for a further 24 hours (4/24/2 assays). Four duplicate cultures were used for each point within a replicate. Data are fitted to a second-order (quadratic) equation. Dotted lines are extrapolations to the horizontal axis calculated from the quadratic plots. Axes are truncated and some symbols are excluded to improve clarity, especially around the origin. **A**. SHIV_SF162P3_ exposed to GHOST cells from passage 7 (1 replicate) and 9 (2 replicates). Gray: control cultures where virus were incubated without monoclonal antibody: y = -0.00285 x^2^ + 1.310 x -6.009; green: Virus pre-incubated with 0.625 µg/ml IgG1 b12: y = -0.00284 x^2^ + 0.939 x -0.517. **B**. Gray same as for A. blue: Virus pre-incubated with 0.25 µg/ml IgG1 b12: y = -0.000606 x^2^ + 0.870 x + 3.152. **C**. SHIV_SF162P3_ exposed to GHOST cells from passages 15, 17 and 21. Gray: control cultures where virus were incubated without monoclonal antibody: y = 0.00182 x^2^ + 0.665 x + 11.01; green: Virus pre-incubated with 0.625 µg/ml IgG1 b12: y = + 0.00135 x^2^ + 0.487 x + 8.334. **D**. Gray same as for C. blue: where cultures are exposed to virus pre-incubated with 0.25 µg/ml IgG1 b12: y = 0.00140x^2^ + 0.616x + 5.768. Interval between points where control and 0.25 µg/ml IgG1 b12 plots cut x-axis: 7.81 infectious virus.

SHIV_SF162P3_ is more resistant to neutralization than the parental HIV-1 strain or the SHIV_SF162P4_ variant (Figure 8) requiring higher concentrations of monoclonal antibody to produce reductions in infectivity. With early passage cells and 80 infectious doses of virus, there is 30% neutralization with 0.625 µg/ml IgG1 b12 (Figure 8A) and 14% with 0.25 µg/ml (Figure 8B). At higher passage levels and the same dose of virus, neutralization is 26% with 0.625 µg/ml IgG1 b12 (Figure 8C). At the lower dose of antibody, the plots are close to parallel: neutralization was 16% with 80 infectious doses. The interval between the points where the plots cross the horizontal axis is equivalent to eight infectious doses.

The IgG1 b12 monoclonal antibody neutralizes both SHIV _SF162_ variants and the parental, primary HIV-1 _SF162_ isolate. However, inactivation is slow and requires relatively high concentrations of antibody especially with the resistant variant. Inactivation continues after the virus-antibody mixture is exposed to the target cells so that the proportion of inactivated virus varies with the size of the inoculum. The remarkable feature is that complete inactivation of a small dose of virus can occur with a relatively low concentration of antibody although this also depends on the surface density ratio of the CD4 receptor : CCR5 co-receptor on the target cells.

## Discussion

Results from *in vitro* neutralization assays are not a reliable guide to *in vivo* protection [44]: rhesus macaques with relatively high concentrations of serum neutralizing antibodies are not necessarily protected from SHIV challenge. The discrepancies may be attributed to a number of sources: the extent of inactivation when a virus forms a complex with an antibody, whether the antibody can produce an all or nothing loss in virus infectivity or only reduces the rate of a reaction associated with viral pathogenicity; the properties of the assay target cell may be different to those of the first cell to be infected *in vivo*; the size and nature of the inoculum. Various scenarios can be proposed to reconcile the conflicting observations. Where the *in vitro* neutralizing properties of the antibodies approximate those in the traditional concept of neutralization and the virus is relatively sensitive to neutralization there is a correlation between *in vitro* and *in vivo* results irrespective of the dose of virus [44]. Alternatively, protection may be seen if antibodies which cannot fully inactivate the virus are present in sufficient concentrations to reduce the replication of relatively resistant virus to levels below the detection limit of the laboratory assays. It is also possible that low concentrations of antibody may be able to completely inactivate neutralization resistant virus but only at low infectious doses [44]. It may be recalled that protection in rhesus macaques against a repeated low dose challenge with SIV_smE660_ correlated with low levels of *in vitro* neutralization [45].

The human monoclonal IgG1 b12 is both protective in the SHIV_SF162_ challenge model with rhesus macaques as well as neutralizing in *in vitro* assays [13–17]. There are exceptions and anomalies within this scenario. For example, a macaque that received 1 mg/kg of IgG1 b12 was infected following six repeated challenges with 3 TCID_50_ SHIV_SF162P3_ while control macaques remained uninfected [15]. This may be attributed to experimental variation. Alternatively, IgG1 b12 may not fully inactivate virus. In an earlier study, protection following immunization of macaques was associated with sera whose neutralization rates were the same at different antibody concentrations during the absorption phase. Macaques with sera whose neutralization-absorption rates were different were not protected [44]. IgG1 b12 follows the latter pattern: in the present study (Table 1 and Figure 2), the rates of neutralization with HIV-1_SF162_ during the absorption phase of a GHOST neutralization assay varied with the IgG1 b12 concentration. Alternatively, the discrepancies may be attributed to anomalies in the quantification of the variables. It is possible to titrate the challenge virus both *in vivo* and *in vitro*: 10 TCID_50_ may be equivalent to 2 monkey infectious doses (MID_50_) in a random sample of macaques. However, the same dose may represent 5 MID_50_ in the more sensitive macaques but only 1 MID_50_ in the more resistant. If the transfer of monoclonal antibody were protective against 4 MID_50_, the resistant macaques would be protected while the sensitive macaques will eventually succumb to infection. Similarly, transfer of high doses of IgG1 b12 may be sufficient to produce a pharmacological effect, slowing virus replication below the level of detection without necessarily protecting against infection.

The target cells appear to play an active role in HIV neutralization [46]. Virus binds to CD4 molecules on the cell surface and then to its co-receptor [47]. It is possible that there is a delay between the first and second steps which depends on the availability of the CCR5 molecule. A low density of CCR5 molecules on the cell surface may increase any delay allowing the antibody reaction more time to influence the infectious process. In the present study the density of CCR5 on the cell surface did influence neutralization at the lowest doses of virus. In addition, it is possible that the cells’ role in neutralization may involve antibody binding with one arm to the envelope glycoprotein on the virus particle and then, with its other arm, to a host-derived protein either on the virus or on the target cell [48]. This is possible since several neutralizing antibodies to HIV-1 are cross-reactive with host proteins. However, this is unlikely to be the case with IgG1 b12 since this antibody shows little polyreactivity. For IgG1 b12, the kinetics of neutralization may reflect the conformational changes of the HIV-1 envelope glycoprotein. There are only small changes in entropy when IgG1 b12 binds to the HIV-1 envelope but major changes follow binding to CD4 on the target cell [49]. Any IgG1 b12 molecules which have bound to the virus may then be in a position to complete inactivation.

In our previous studies with peripheral blood mononuclear cell (PBMC) neutralization assays we were able to distinguish between two different scenarios: a percentage reduction in virus dose against an absolute reduction in virus dose; complete inactivation and reductions in virus replication [46,50]. In conventional assays where a virus inoculum of 50-200 TCID_50_ is used, if there is a 50% reduction in the production of HIV-1 Gag p24 antigen, this does not distinguish between half of the virus being inactivated or none of the virus being fully inactivated but with a 50% reduction in virus replication. In our assays, the virus was diluted in series and each dilution was mixed with antibody before exposure to target cells. Virus was titrated in this way with control and experimental antibodies. If an inoculum of virus with 5 infectious doses were reduced to a single dose, this would represent 80% neutralization. However, it could also be described as complete inactivation of 4 TCID_50_. If virus were completely inactivated this would be reflected in a reduction in virus titer. If antibody only induced a lower replication rate, any early difference in virus titers would be lost as the culture phase was extended [46]. Using these PBMC assays, we were able to demonstrate subtype-associated neutralization [50]. Using dendritic -T-cell co-cultures – which might reflect the situation in natural transmission - higher numbers of infectious virus were inactivated than with PBMCs as target cells [51]. Also, sera from immunized macaques neutralized primary isolates of HIV-1 [51].

In the traditional view of neutralization the target cells are passive recorders of the infectious virus dose remaining at the end of the incubation phase. In HIV neutralization of primary isolates, the target cells appear to take an active role [46]. Antibodies do bind to the free virion. This conclusion follows from the observation that neutralization increases with the time allowed for antibody to interact with free virions (Figure 2). One hypothesis is that the virus requires multiple antibodies to bind for it to lose infectivity. This deduction is based on the observation that inactivation during the incubation phase in double plots is not linear (Figure 3). An alternative hypothesis is that antibody binds to virus but the complex remains infectious until it is exposed to target cells; the nature of the antibody and the receptor determine whether virus is completely inactivated, has a reduced replication rate, there is no effect or even enhancement [52,53]. In the present study we have assumed that the absorption plots are linear. However, this may not be so. If neutralization during the absorption phase is non-linear, the plots may extrapolate back to zero so that there would be no loss of virus infectivity during the incubation phase. Under these circumstances, the apparent increased neutralization seen with extended incubation phases would represent the propensity of a virus to be inactivated following exposure to target cells. The GHOST assay is not sufficiently precise to distinguish between these alternatives.

GHOST cell assays offer the advantage of quantifying the number of infected cells within individual cultures. However, the initial assumption that a fluorescent GHOST cell represents infection with a single virus may only be accurate at the lowest doses. The plot of virus dilution against the number of fluorescent cells is only linear over a restricted range of virus doses. At higher doses, the data fit a quadratic plot better than a straight line (compare Figure 1A with 1B). In a quadratic equation, the fitted plot includes a term where the number of infectious doses is multiplied by itself. At least two scenarios are possible, depending on the homogeneity of the virus: at low doses, a single infectious virus may be able to stimulate a cell to produce enough fluorescent protein to be detectable in a scanner. The increase in the number of fluorescent cells which is seen with higher viral doses may be linear up to an inoculum of 100-200 virus for HIV-1; at higher doses, cells are exposed to multiple virus particles each of which may not be sufficient to induce enough fluorescence to be detectable but they can do so in combination. Under these conditions, the increase in the number of fluorescent cells will not be directly proportional to the virus dilution and the quadratic curve will hide the linear plot with these higher inocula. Alternatively, the quadratic curve may be continuous and all cells need to be infected by multiple virus particles to produce sufficient fluorescence for detection. The precision of the current assay is not sufficient to distinguish between these hypotheses. In the present study, there are approximately 3,200 virus particles for each infectious dose of HIV-1 _SF162_ in GHOST cells (Table S1). If we examine 10,000 events, where there are 10 infectious virus doses, the cells will be exposed to an inoculum of 32,000 virus particles: assuming a Poisson distribution and all virus particles have the same probability of binding only 400 cells will not encounter a single virus particle. Most cells (83%) will be exposed to more than one virus particle.

The capacity to confidently predict the outcome of a rhesus macaque challenge study, or a Phase III efficacy trial in humans, remains elusive. A practical approach would be to use sera from immunized individuals in successful trials to test a range of different assays. The variables which seem likely to influence any correlation of *in vitro* and *in vivo* results are: whether the critical events involve an interaction between antibody and free virion or antibody and cell-associated virus; whether a decrease in absolute number of infectious virus or the proportion of inoculum is important; whether antibody produces an all or nothing loss of infectivity or a reduction in viral replication rate. These factors may also vary with the route of infection involved in natural transmission. Assays with GHOST cells may be useful in distinguishing between an absolute decrease and a proportional reduction while PBMCs would allow monitoring of the replication of a single infectious dose of virus [46,50]. The immediate concern is to be able to demonstrate any *in vitro* neutralization of the more resistant HIV-1 isolates with these sera. In a previous study we have demonstrated neutralization of HIV-1 _Han2_ in GHOST cell assays with extended incubation phases and sera from people immunized with recombinant canary pox virus expressing HIV-1 _MN_ gp120 and boosted with recombinant HIV-1 _SF2_ gp120 [54]. HIV-1 _Han2_ was moderately resistant to neutralization by plasma from HIV-1 seropositive people. We suggest that passive transfer studies in the SHIV challenge rhesus macaque model should be used to quantify protection at different doses of virus and concentrations of antibody. These results should then be compared with equivalent *in vitro* assays to determine a correlate of protection.

Our hypothesis is that neutralizing antibodies can only completely inactivate a small number of infectious primary HIV-1 doses both *in vitro* and *in vivo*. Standard neutralization assays are unable to detect this effect. Assay conditions have to be modified to magnify the effect

## Materials and Methods

### Ethics statement

Virus stocks were prepared in peripheral blood mononuclear cells (PBMCs) derived from humans or rhesus macaques. The two rhesus macaques (*Macaca mulatta*) used in this study were captive bred for research purposes and were socially housed at the BPRC. BPRC facilities comply with Dutch law on animal experiments (Wet op de Dierproeven and its adaptations as published in the Staatscourant), the European Council Directive 86/609/EEC, as well as with the ‘Standard for humane care and use of Laboratory Animals by Foreign institutions’ identification number A5539-01, provided by the Department of Health and Human Services of the United States of America’s National Institutes of Health (NIH). Enrichment was provided in the form of pieces of wood, mirrors, food puzzles, a variety of food and other home made or commercially available enrichment products. Animals were fed with standard food pellets, fruit and bread. Water was provided *ad libitum*.

Animals were monitored daily for health and discomfort. The Institutional Animal Care and Use Committee (BPRC Dier Experimenten Commissie, DEC) pre-approved all procedures. The qualification of the members of this committee, including their independence from a research institute, is requested in the Wet op de Dierproeven (1996). At the BPRC all animal handling is performed within the Department of Animal Science (ASD) according to Dutch law. A large experienced staff is available including full time veterinarians and a pathologist. The ASD is regularly inspected by the responsible authority (Voedsel en Waren Autoriteit, VWA) and an independent Animal Welfare Officer. All steps were taken to ameliorate the welfare and to avoid any suffering of the animals. Animals were sedated with ketamine before blood was taken. Neither of the animals from which peripheral blood was obtained was used exclusively for this purpose, in full accordance with the 3Rs, reducing the numbers involved in animal experiments. No animal was sacrificed during the course of these studies. The Council of the Association for Assessment and Accreditation of Laboratory Animal Care (AAALAC International) has awarded full accreditation to the BPRC. Thus, the BPRC is fully compliant with international demands on animal studies and welfare as set out by the European Convention for the Protection of Vertebrate Animals used for Experimental and other Scientific Purposes, Council of Europe (ETS 123 including the revised Appendix A), Dutch implementing legislation and the Guide for Care and Use of Laboratory Animals.

At the time of their donation, human volunteers are invited to sign a form indicating that they are willing for their blood to be processed for scientific purposes. Buffy coats were provided to the BPRC anonymously: the source of the blood cannot be identified and neither personal nor clinical details of the donors are available.

### Virus isolates

Primary HIV-1_SF162_ (original donor: J. Levy [19]) was obtained from the AIDS Research and Reference Reagent Program, Division of AIDS, NIAID, NIH, Washington DC, USA. The stock was prepared in phytohemagglutinin-transformed, recombinant human interleukin-2 maintained human PBMCs. Human PBMCs were donated by volunteers to the Stichting Sanquin Bloedvoorziening, Rotterdam.

SHIV_SF162_ is a chimeric virus constructed with the envelope glycoproteins of HIV-1_SF162_ and the internal structural proteins and enzymes required for viral replication of simian immunodeficiency virus clone SIV_mac239_ [7]. The virus was passaged rapidly through rhesus macaques four times (SHIV_SF162P4_) [12]. After the third passage one macaque became ill and SHIV_SF162P3_ was isolated 21 weeks after infection. SHIV_SF162P4_ was obtained from Dr. Leonidas Stamatatos [11]. SHIV_SF162P3_ was obtained from Dr. Cecilia Cheng-Mayer [8].

### Preparation of SHIV stocks

SHIV stocks were prepared in rhesus macaque PBMCs cultured on feeder cells [55]. After their irradiation with 25 Gy, 5 x 10^5^ ADP / BSM cells (Epstein–Barr virus transformed human B-lymphocytes) and 2 x 10^6^ human PBMCs were seeded two hours prior to the addition of rhesus macaque PBMCs. The rhesus PBMCs were taken from a macaque which was selected for higher *in vitro* virus replication and depleted of CD8 T cells with magnetic beads. The mixture of cells was cultured for six days in RPMI medium with 10% (v: v) rhesus macaque serum and phytohemagglutinin. Virus was then added to the mixture and replication monitored by production of SIVgag antigen. Virus was harvested when the SIV antigen reached its peak.

### Monoclonal antibody

The human IgG1 b12 monoclonal antibody was obtained through the NIH AIDS Research and Reference Reagent Program, Division of AIDS, NIAID, NIH from D. Burton of the Scripps Institute, La Jolla, California, USA [13].

### Cells

GHOST(3) Hi-5 cells are human osteosarcoma cells which have been engineered to express the CD4 receptor and green fluorescent protein following infection with HIV-1 or SHIV. The cell line was obtained through the NIH AIDS Research and Reference Reagent Program, Division of AIDS, NIAID, NIH from Dr. Vineet N. KewalRamani and Dr. Dan R. Littman [41]. The cells have been engineered and selected for high expression of CCR5, the co-receptor for the HIV-1 isolates used in this study. The cells were passaged in Dulbecco’s Modified Eagle Medium with 10% (v: v) fetal calf serum but without further selection. The culture from the aliquot received from the repository was deemed to be passage 1. A master stock was prepared at passage 3 and working stocks were prepared at passage 5. Cells were passaged twice weekly from the working stock.

### Neutralization assays

All neutralization assays are described as a/b/c where a is the time in hours (= incubation) during which antibody and virus are incubated prior to exposure to target cells (= absorption) for b hours. The cells are then washed and incubated for c days (= culture). The culture phase is timed form the cells’ first exposure to virus. All three incubations are at 37°C. All sera were heat inactivated at 56 ^o^C for one hour.

The number of individual infectious events can be quantified in GHOST cells using a fluorescent activated cell scanner. For neutralization assays a fixed dilution of each virus stock was chosen based on the results of a previous titration: for neutralization kinetics studies the virus dilution was chosen to give between 200 and 3,000 fluorescent cells per 10,000 recorded events. At the higher doses some cells are infected with more than one infectious virus. The dose of virus was adjusted in accordance with the Poisson distribution. One hundred and ninety µls of the fixed virus dilution were incubated for a given interval (= a hours) with 10 µls of a serum dilution at 37 °C. The virus-antibody mixture was added to GHOST cells which had been seeded 24 h previously at 6 x 10^4^ cells per well in 24-well cell culture plates (Greiner bio-one #662160, Kremsmunster, Austria). After an absorption period (= b hours) the cultures were washed once and cultured for a total of two days (= c), i.e. the culture period is timed from the first exposure of the cells to the virus. Note that no additives are used to enhance virus binding to target cells. Subsequently, the cells were removed from the plastic by 1 mM EDTA and fixed in paraformaldehyde at a final concentration of 2%. The cells were analyzed with a FACSsort® flow cytometer (Becton Dickinson). The living cells were gated on the basis of forward and side scatter. Using these parameters, uninfected cells were further gated on fluorescence to set the upper limit of the region. The number of infected cells was then determined using the gates with the uninfected cells. The virus titer following incubation with antibody is divided by its titer following incubation as free virus and plotted on a log scale against the incubation (a) or absorption (b) time [43,54].

The position of each virus – antibody combination was randomized on the plates so that 20 combinations with four controls could be tested in a single experiment. There were four duplicate plates. To reduce variation, the plates were boxed in pairs and incubated along side each other rather than stacked vertically.

### FACS analysis of GHOST cells

Phenotypic analysis of Hi-5 GHOST(3) cells following passage was performed by fluorescence-activated cell scanning analysis. For multi-color staining, 50 to 100 µl of cells were incubated with 25 µl of the monoclonal antibody mix for 15 min at room temperature. The cells were centrifuged for 10 min at 200 × *g*. Supernatant was aspirated, and the cells were resuspended in phosphate-buffered saline and fixed in paraformaldehyde.

Flow cytometry was performed on a FACS ARIA using the DIVA software (Becton Dickinson). Monolayers of Hi-5 GHOST(3) cells were treated with EDTA. Single cells were gated on the basis of forward and side scattering. At least 10,000 events were analyzed. Antibodies against CD4 conjugated to R-phycoerythrin and a cyanine dye (Cy7) (Becton Dickinson, Lincoln Park, N.Y.) and the 2D7 monoclonal antibody (Pharmigen, Woerden, The Netherlands) against CCR5 and conjugated to phycoerythrin-A were used.

### Mycoplasma test

GHOST cells from different passage levels were tested for mycoplasma using a polymerase chain reaction assay with Mycoplasma primer set A (Bioo Scientific, Austin, Texas, USA) according to the manufacturer’s recommendations.

### Viral RNA determinations

The number of virus particles with RNA was determined using an adapted version of a published SIV-gag-based real-time PCR assay [56]. The SIV-probe used was identical to the probe described [56] except that we used the quencher dye Black Hole Quencher 2 instead of TAMRA. The forward (SIV31) and reverse (SIV41) primers were essentially identical to primers SIV.510f and SIV.592r [56], with minor modifications to improve the sensitivity of the assay. The SIV31 and SIV41 primer sequences were 5′-CCAGGATTTCAGGCACTGTC-3′ and 5′-GCTTGATGGTCTCC CACACA-3′, respectively. The PCR was carried out using the Brilliant® QRT-PCR Core Reagent Kit, 1-Step (Stratagene, Europe, Amsterdam, The Netherlands) in a 25 µl volume with final concentrations of 160 nM for each primer, 200 nM for the probe, 5.5 nM MgCl_2_, and using 10 µl RNA. RNA was reverse transcribed for 30 min at 45°C. Then, after a 10 min incubation step at 95°C, the cDNA was amplified for 40 cycles, consisting of 15 s denaturation at 95°C, followed by a 1 min annealing-extension step at 60°C. All the reactions were carried out with an iQ™5 Multicolor Real-Time PCR Detection System (Bio-Rad Laboratories BV, Veenendaal, The Netherlands). Detection limit is 100 RNA copies/ml.

### Statistics

Statistical analyses were performed using GraphPad Prism version 5.0d for Windows, GraphPad Software, San Diego, California, USA, www.graphpad.com. All calculations were performed to four significant figures and then adjusted to three significant figures or three decimal places where appropriate. Regression coefficients and probability values are given to four significant figures. Linear regression lines are recorded as y = mx + c where m is the gradient and c the intercept (the value of y when x = 0). Equations for data fitted to a second-order or quadratic plot are recorded as y = lx^2^ + mx + c. The coefficient of determination (r^2^) gives the proportion of the variability in the dependent variable which can be attributed to the independent variable.

Neutralization rates (Figures 2 and 3 and Table 1): The rate of neutralization with primary isolates of HIV-1 is relatively slow in comparison to other viruses. We chose therefore to present neutralization rates in terms of log_10_ reductions in infectious virus titer per hour rather than the customary log_e_ reductions per second. Plots are presented as the regression line with its 95% confidence band.

Plots of virus dose against numbers of fluorescent cells were analyzed either by linear regression or data were fitted to a second-order plot (Figures 1, 7 and 8). The gradients for virus in control cultures or following incubation with IgG1 b12 were then compared. If there was no significant difference between the gradients, a gradient was calculated from the pooled data and the resulting intercepts of the plots compared.

## Supporting Information

Table S1 **Titers in GHOST cell cultures and RNA copy number of stock virus.**
(DOC)
