# Supplementary material for: In Vitro Neutralization of Low Dose Inocula at Physiological Concentrations of a Monoclonal Antibody Which Protects Macaques against SHIV Challenge
Source: PLoS One. 2013 Aug 16;8(8):e72702. doi: 10.1371/journal.pone.0072702 (PMC3745472; doi:10.1371/journal.pone.0072702)
Supplement: Table S1 — Titers in GHOST cell cultures and RNA copy number of stock virus. (DOC) [file pone.0072702.s001.doc]

Supplementary Table 1: Titers in GHOST cell cultures and RNA copy number of stock virus.

| **Virus** | **PBMC** | **High Five** | **PCR** | **Particle : Infectious Ratio** |
| --- | --- | --- | --- | --- |
| HIV-1 _SF162_ | human | 2.27 x 10 ^5^ / ml* | 1.45 x 10 ^9^ / ml ^§^ | 3.19 x 10 ^3^ |
| SHIV_SF162P4_ | rhesus | 1.24 x 10 ^5^ / ml | 1.00 x 10 ^9^ / ml | 4.03 x 10 ^3^ |
| SHIV_SF162P3_ | rhesus | 1.46 x 10 ^4^ / ml | 4.00 x 10 ^8^ / ml | 1.36 x 10 ^4^ |

^*^ Tissue culture infectious doses per ml (= number of fluorescent cells)

^§^ HIV virus particles (= RNA copies divided by two)
